# Comparison between the safety of the HPV vaccine versus placebo: a systematic review and meta-analysis of randomized clinical trials

**DOI:** 10.1016/j.jped.2025.04.009

**Published:** 2025-06-06

**Authors:** Swelen Aparecida dos Santos, Mariane Yoshie Sato, Pedro Henrique Gunha Basilio, Meire Ellen Pereira, Rafaela Climaco Julião, Nielson da Cunha Arruda, Davi Paula da Silva, Cláudia Sirlene Oliveira, Victor Horacio de Souza Costa-Junior, Izonete Cristina Guiloski

**Affiliations:** a Instituto de Pesquisas Pelé Pequeno Príncipe, Curitiba, PR, Brazil; b Faculdades Pequeno Príncipe, Curitiba, PR, Brazil; c Unicesumar, Curitiba, PR, Brazil; d Hospital Pequeno Príncipe, Curitiba, PR, Brazil

**Keywords:** Papillomavirus infections, Immunization, Uterine cervical neoplasms

## Abstract

**Objective:**

Human Papillomavirus (HPV) is a virus that targets epithelial tissues. Virtually all cases of cervical cancer are related to HPV, emphasizing the importance of vaccines in prevention. Although >200 million doses have been administered worldwide, concerns persist about adverse reactions. This study evaluated the safety of the HPV vaccine and the main adverse effects.

**Data sources:**

The study was registered in the PROSPERO database (CRD42023365692). The systematic searches were conducted in the PubMed, Scopus, Embase, Cochrane, Science Direct, and Web of Science databases using the search strategy "HPV" AND "vaccine" AND "safety" NOT "COVID" from 01/01/2007 to 31/12/2022. Inclusion criteria were based on the PICOT strategy, focusing on studies with humans, vaccinated populations comprising children, adolescents, and adults, and Phase II/III randomized clinical trials. The PEDro scale was used to assess the quality of the studies.

**Summary of findings:**

Eleven articles were qualified for qualitative synthesis and meta-analysis. The results indicated that HPV vaccination was associated with increased local reactions, fatigue, and myalgia compared to the placebo. However, there were no significant differences in serious adverse events, gastrointestinal reactions, cutaneous effects, headache, or fever between the vaccine and placebo groups.

**Conclusion:**

Local reactions, fatigue, and myalgia were more prevalent in the HPV vaccine group; the overall safety profile of the vaccine was favorable. The HPV vaccine was deemed safe, mirroring the profile of adverse reactions seen with other vaccines. With its potential to prevent cancer, the benefits of HPV vaccination far outweigh the minimal risks.

## Introduction

Human Papillomavirus (HPVs) are part of the Papillomaviridae family. These viruses have a high specificity for tissues and infect both cutaneous and mucosal epithelium. At least 14 of them pose a high risk of causing cancer. HPV types 16 and 18 are responsible for most cancer cases, contributing to approximately 70 % of cervical cancers and precancerous cervical lesions.^1^ Cells infected with high-risk HPV experience an increase in their proliferation rate, and if not controlled by the immune system, they can evolve into precancerous changes or tumors over time.^2^

Several factors, such as prolonged use of oral contraceptives, multiple pregnancies, smoking, a compromised immune system, and co-infection with other sexually transmitted diseases, in addition to the specific type of HPV (high-risk), can increase the chance of developing precancerous cervical cells.^2^ It is important to highlight that virtually all cases of cervical cancer are related to HPV infection. Additionally, HPV infection may also be associated with other types of cancer, such as anogenital (vulvar, vaginal, anal, and penile) cancer,^3^ head and neck cancer,^4^ and the development of genital warts in both men and women.^5^

Six prophylactic HPV vaccines have been licensed, all based on the L1 major capsid antigen, which self-assembles into virus-like particles. Three bivalent vaccines protect against the oncogenic types HPV-16 and HPV-18: Cervarix® (GSK), Cecolin® (Innovax), and Walrinvax® (Walvax/Zerun). Two quadrivalent vaccines protect against the low-risk types HPV-6 and HPV-11, in addition to the high-risk types HPV-16 and HPV-18: Gardasil® (MSD) and Cervavac® (Serum Institute of India). The nonavalent vaccine Gardasil 9® (MSD) protects against infections caused by HPV types 6, 11, 16, 18, and five additional high-risk types: 31, 33, 45, 52, and 58.^6^, ^7^, ^8^

So far, >200 million doses of preventive HPV vaccines have been administered globally.^9^ There is a growing body of evidence confirming the safety of these vaccines. However, some safety issues have emerged, such as the occurrence of pain, redness, or swelling at the injection site, fever, headache, fatigue, nausea, and myalgia. Although such reports have surfaced, serious adverse events are extremely rare following vaccination with HPV vaccines,^10^ and there is no difference in specific adverse effects among Cervarix®, Gardasil®, and Gardasil 9® vaccines.^11^ Other vaccines, such as those against influenza, hepatitis B, and COVID-19, also present these adverse effects, suggesting this to be a widespread and nonspecific occurrence among vaccines.^12^

Given the importance of HPV vaccination in preventing infection and cancer occurrence, this study aimed to evaluate through a systematic review and meta-analysis "How safe are HPV vaccines and what are the main adverse reactions for healthy individuals?"

## Methodology

### Search strategy

The study was conducted according to the recommendations of the PRISMA guideline. The review protocol was registered in the PROSPERO database (CRD42023365692). The systematic search was implemented in PubMed, Scopus, Embase, Cochrane Library, Science Direct, and Web of Science. The search strategy “HPV” AND “vaccine” AND “safety” NOT “COVID”. Two researchers (MYS, PHGB) independently selected the articles, and conflicts were discussed with a third author (SAS).

### Eligibility criteria

The inclusion criteria were based on the research question: "How safe are HPV vaccines and what are the main adverse effects?" To establish such criteria, the PICOT strategy (Population, Intervention, Control, Outcome, Type of Study) was used. The studied population consisted of healthy individuals. The intervention was HPV vaccination. Comparison was made with individuals who received a placebo, and the primary outcome was to analyze the safety and major adverse reactions post-vaccination. For the type of study, the authors considered Phase II/III Randomized Clinical Trials.

Eligibility criteria were a) Articles published in English; b) Articles involving human subjects; c) Vaccinated population consisting of children, adolescents, and adults; d) Abstracts available in selected databases; e) Articles relating to the vaccine and HPV; f) Vaccine safety; g) adverse effects of HPV vaccine; h) Healthy individuals and non-pregnant women; i) Phase II/III randomized clinical trials; j) Articles between 2007 and 2022. Articles that did not match the eligibility criteria were excluded from the selection.

### Data extraction and qualitative synthesis

Three independent authors (PHGB, MS, and RJ) extracted information from each article and presented it in a spreadsheet containing title, DOI, access link, authors, year, journal, country, study type, participants, study demographics: number of participants, age range; study characteristics: type of control, primary outcome, a secondary outcome, and follow-up time; vaccine data: HPV vaccine type, number of doses, the interval between doses, days after vaccination for symptoms, local reactions, injection site pain, swelling at the injection site, redness at the injection site, stiffness, sweating, malaise, fatigue, fever, headache, arthralgia, myalgia, rash, urticaria, gastrointestinal symptoms, respiratory symptoms, dizziness, genitourinary tract symptoms, the onset of chronic diseases, the onset of autoimmune diseases, vaccine-related systemic effect, serious adverse event, death.

### Quality of the studies

To evaluate the quality of the studies, the PEDro scale (Physiotherapy Evidence Database) was used. The instrument was applied in the articles selected for meta-analysis and consists of 11 items. Each article receives a score according to the PEDro Quality Scale, ranging from 0 to 10, one point for each criterion, except for the first, since it concerns the external validity of the study, not entering the actual score. Thus, the more points an article has, the lower the risk of it containing biases: from 0 to 4 points is considered a low-quality article; 5 and 6, intermediate; and from 7 to 10, high quality.^13^

### Meta-analysis

The authors performed the meta-analysis using vaccine-related adverse events (local reaction, fever, headache, fatigue, cutaneous symptoms, myalgia, and gastrointestinal symptoms) as dichotomous variables, which were analyzed by the Mantel-Haenszel method, and a random-effects model was applied. Results from random-effects models were reported as relative risks (RR) with 95 % CI. Heterogeneity was assessed by the I-square (I^2^) index and ranked as: low heterogeneity (< 25 %), mild heterogeneity (25–50 %), moderate heterogeneity (50–75 %), and high heterogeneity (> 75 %).^14^

Funnel plots were also employed to evaluate potential study bias, and subgroup analysis was performed using HPV vaccine valence (bivalent or quadrivalent) as a grouping variable. The statistical significance level was set at *p* < 0.05. All analyses were performed using Review Manager version 5.4.1 (Cochrane Collaboration).

## Results

**Figure. 1:**
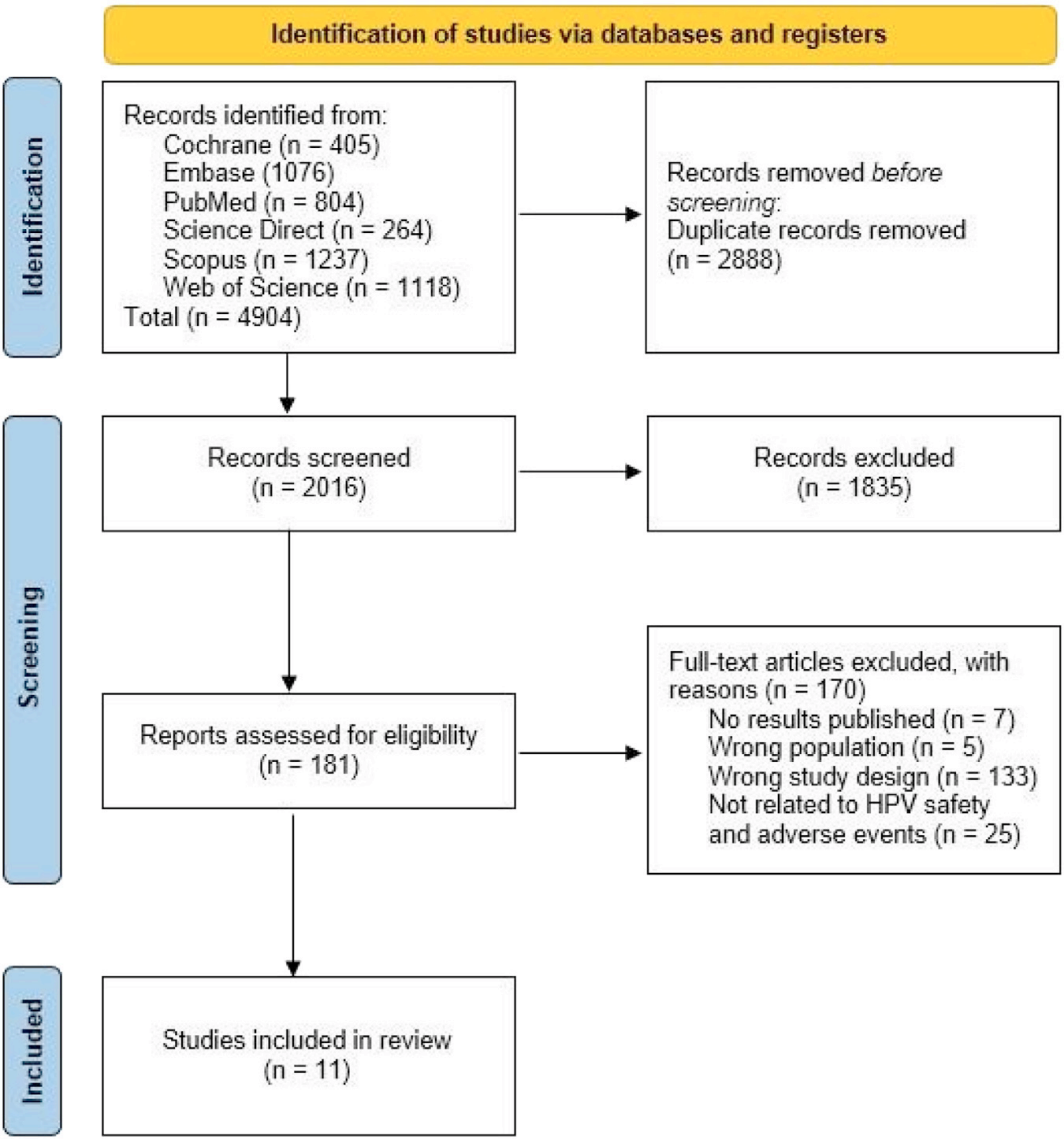
Prisma Flowchart demonstrating the article selection steps.

A total of 4904 articles were selected from different databases: 405 articles from Cochrane Library, 1076 from Embase, 804 from PubMed, 264 from Science Direct, 1237 from Scopus, and 1118 from Web of Science. Subsequently, 2888 duplicate articles were excluded. The remaining 2016 documents were screened based on their titles and abstracts. Based on the eligibility criteria, 181 articles were selected for full-text assessment. In the end, 11 articles were included in the qualitative synthesis and meta-analysis (Figure. 1).

In the present study, 11 articles published between 2007 and 2019 met the eligibility criteria. Of the 11 articles, four have their country of origin in China, while the remainder are from countries in Europe, Asia, Africa, and America (Table 1).

**Table 1:** Characteristics of the selected studies.

Reference	Country	Participants characteristics Sex/Age^a^ (*n*)	Vaccine/ Number of doses/ Application range^b^	Results
Chen et al.^15^	China	Females 20–45 (1499)	Quadrivalent Gardasil 3 doses 0–2–6	Four participants (two in the qHPV vaccine and two in the placebo) discontinued the study vaccination due to adverse effects (AEs) that were considered vaccination-related. Within 15 days following any vaccination, injection-site AEs were more frequent among qHPV vaccine recipients, and systemic AEs were similar in frequency between the qHPV vaccine and placebo groups.
Kim et al.^37^	South Korea	Females 15–25 (140)	Bivalent Cervarix 3 doses 0–1–6	Local (pain) and general (fatigue, myalgia, or headache) symptoms were commonly reported in both groups.
Li et al.^19^	China	Males 9–15 (100) Females 9–45 (500)	Quadrivalent Gardasil 3 doses 0–2–6	The qHPV vaccine was generally well tolerated. Injection site AEs were higher in vaccine than placebo recipients. Vaccine-related systemic AEs were reported with similar frequency in vaccine and placebo recipients. There was one serious AE among placebo recipients that was determined by the investigator to be not related to vaccination.
Mikamo et al.^11^	Japan	Males 16–26 (554)	Quadrivalent Gardasil 3 doses 0–2–6	Vaccination-related AEs were slightly higher in the qHPV vaccine than in the placebo group. The most common reactions were mild to moderate injection site pain, erythema, and swelling.
Moreira et al.^16^	Australia Canada Colombia Denmark Hong Kong Mexico Sweden United States	Females 12–26 (608)	Nonavalent Gardasil 3 doses 0–2–6	The most common AEs were injection-site events, the majority of which were mild. Overall, the 9vHPV vaccine was generally well tolerated in prior qHPV vaccine recipients.
Mugo et al.^21^	Ghana Kenya Senegal	Females 9–26 (227)	Quadrivalent Gardasil 3 doses 0–2–6	Across vaccination groups, the most common AEs were at the injection site, including pain, swelling, and erythema. No subject discontinued study medication due to an AE and no serious AEs were reported. There were no deaths. This study demonstrated that qHPV vaccination of Sub-Saharan African women was highly immunogenic and generally well tolerated.
Muñoz et al.^38^	Colombia France Germany Philippines Spain Thailand United States	Females 24–45 (1908)	Quadrivalent Gardasil 3 doses 1–2–6	The proportion of women who reported a serious AE on days 1–15 after any vaccination was comparable between the vaccine and placebo groups. Injection-site AEs were mainly responsible for the slight increase in AEs recorded in the vaccine group. There were no vaccine-related serious AEs recorded.
Reisinger et al.^22^	North and Latin America Europe Asia	Males and Females 9–15 (1165)	Quadrivalent Gardasil 3 doses 0–2–6	A higher proportion of vaccine recipients than placebo recipients reported one or more injection-site AEs following any vaccination. Rates of fever were similar between vaccination groups. No serious vaccine-related adverse experiences were reported.
Sow et al.^18^	Sub-Saharan Africa	Females 10–25 (450)	Bivalent Cervarix 3 doses 0–1–6	Injection site pain was the most frequent local symptom in both groups. Local reactions were higher among the vaccine group. The most frequently observed general symptoms in both groups were headache and fever. No participant withdrew owing to AEs. No vaccine-related serious adverse events were reported.
Zhu et al.^17^	China	Females 18–25 (3026)	Bivalent Cervarix 3 doses 0–1–6	Symptoms were generally mild, self-limiting, and of short duration. Injection site symptoms (pain, redness, and swelling) were reported in a numerically higher percentage of subjects in the vaccine group than in the control group. One serious adverse effect (gastrointestinal tract infection) was assessed by the investigator as possibly related to vaccination. Safety outcomes between groups were generally similar.
Zhu et al.^20^	China	Females 9–45 (374)	Bivalent Cervarix 3 doses 0–1–6	The 2vHPV had an acceptable safety profile when administered to healthy Chinese females. The incidence of solicited local symptoms following any dose was generally higher in the vaccine group than in the control group. None of the serious AEs was considered to have a causal relationship to vaccination by the investigator, and no event had a fatal outcome.

AEs, adverse effects; HIV, Human immunodeficiency virus; HPV, Human papillomavirus; qHPV, quadrivalent vaccine; 2vHPV, bivalent vaccine; 9vHPV, nonavalent vaccine

a years.

b months.

Regarding gender, nine articles used groups composed solely of women, while two used groups with both men and women. None of the articles were similar in terms of age range, but the youngest age evaluated was 9 years old and the oldest was 45 (Table 1).

Among the 11 articles, four used the bivalent Cervarix vaccine, six used the quadrivalent-HPV Gardasil, and only one utilized the nonavalent-HPV Gardasil vaccine. Unanimously, all articles administered vaccination in three doses. Following the three doses, their intervals were distributed as follows: four articles with an interval of 0–1–6 months; six articles with an interval of 0–2–6 months, and only one with an interval of 1–2–6 months (Table 1).

## Meta-analysis

### Local reaction

**Figure. 2:**
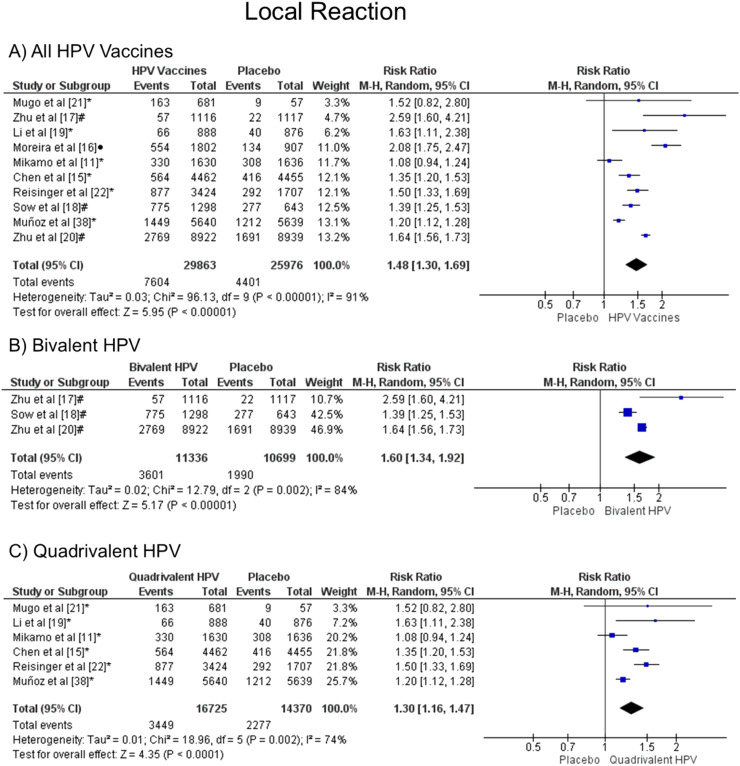
Forest plot of the risk ratio for a local reaction after HPV vaccination. The events indicate how often local reaction effects were reported across the three doses, while the total corresponds to the number of participants multiplied by the number of doses. ^a^bivalent vaccine; ^b^quadrivalent vaccine; ^c^nonavalent vaccine. Funnel plots are presented in Supplementary Figure. 1S.

The injection-site events related to the HPV vaccine were local pain, swelling, and redness. The meta-analysis showed that patients vaccinated with the HPV vaccines have a greater chance of developing local reactions when all vaccine valences are grouped (1.48 [CI: 1.30,1.69]; *p* < 0.00001; I^2^ = 91 %) (Figure. 2a), and when separated by valence, such as bivalent HPV (1.60 [CI: 1.34, 1.92]; *p* < 0.00001; I^2^ = 84 %) (Figure. 2b) or quadrivalent HPV (1.30 [CI: 1.16, 1.47]; *p* < 0.0001; I^2^ = 74 %) (Figure. 2c).

### Fever

**Figure. 3:**
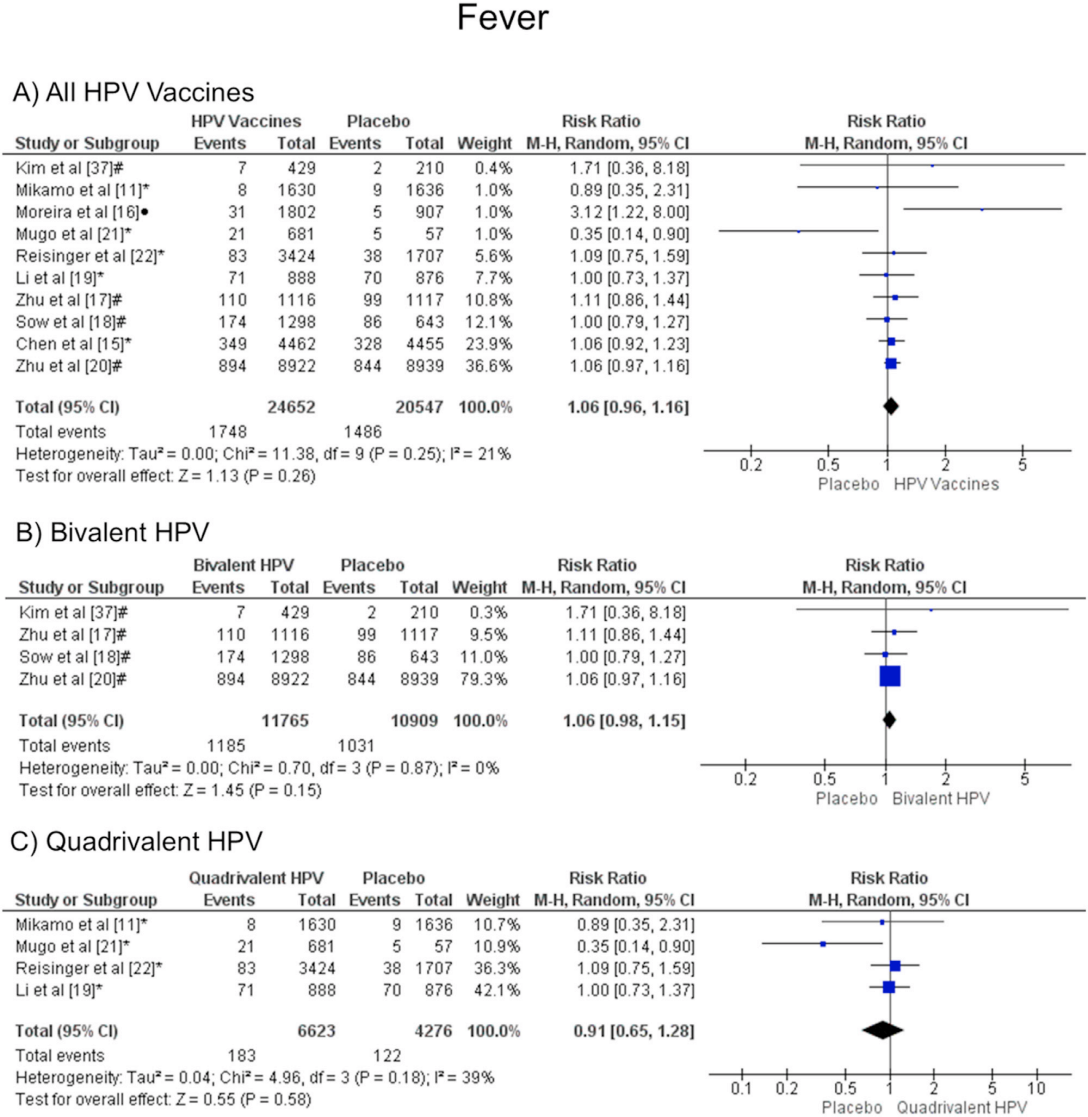
Forest plot of the risk ratio for fever symptoms after HPV vaccination. The events indicate how often fever symptoms were reported across the three doses, while the total corresponds to the number of participants multiplied by the number of doses. ^a^Bivalent vaccine; ^b^quadrivalent vaccine; ^c^nonavalent vaccine. Funnel plots are presented in Supplementary Figure. 2S.

When analyzing fever, there was no statistical difference, both groups are likely to present this symptom, when all vaccine valences are grouped (1.06 [CI: 0.96,1.16]; *p* = 0.26; I^2^ = 21 %) (Figure. 3a), and when separated by valence, bivalent HPV (1.06 [CI: 0.98, 1.15]; *p* = 0.15; I^2^ = 0 %) (Figure. 3b) or quadrivalent (0.91 [CI: 0.65, 1.28]; *p* = 0.58; I^2^ = 39 %) (Figure. 3c).

### Headache

**Figure. 4:**
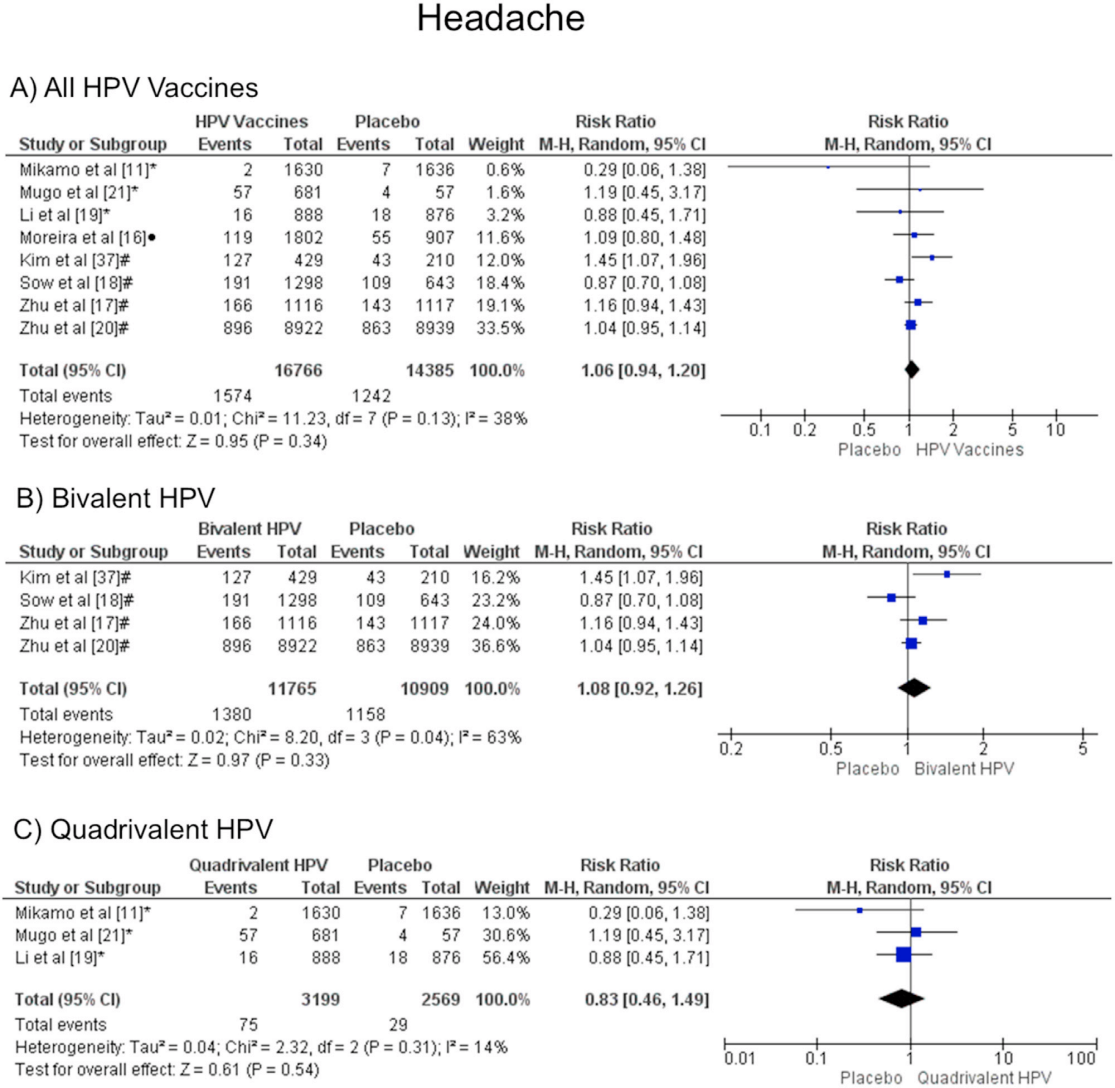
Forest plot of the risk ratio for headache symptoms after HPV vaccination. The events indicate how often headache symptoms were reported across the three doses, while the total corresponds to the number of participants multiplied by the number of doses. ^a^Bivalent vaccine; ^b^quadrivalent vaccine; ^c^nonavalent vaccine. Funnel plots are presented in Supplementary Figure. 3S.

In the headache symptom, there was no statistical difference, both groups are likely to present this symptom, when all vaccine valences are grouped (1.06 [CI: 0.94,1.20]; *p* = 0.34; I^2^ = 38 %) (Figure. 4a), and when separated by valence, bivalent HPV (1.08 [CI: 0.92, 1.26]; *p* = 0.33; I^2^ = 63 %) (Figure. 4b), or quadrivalent (0.83 [CI: 0.46, 1.49]; *p* = 0.54; I^2^ = 14 %) (Figure. 4c).

### Fatigue

**Figure. 5:**
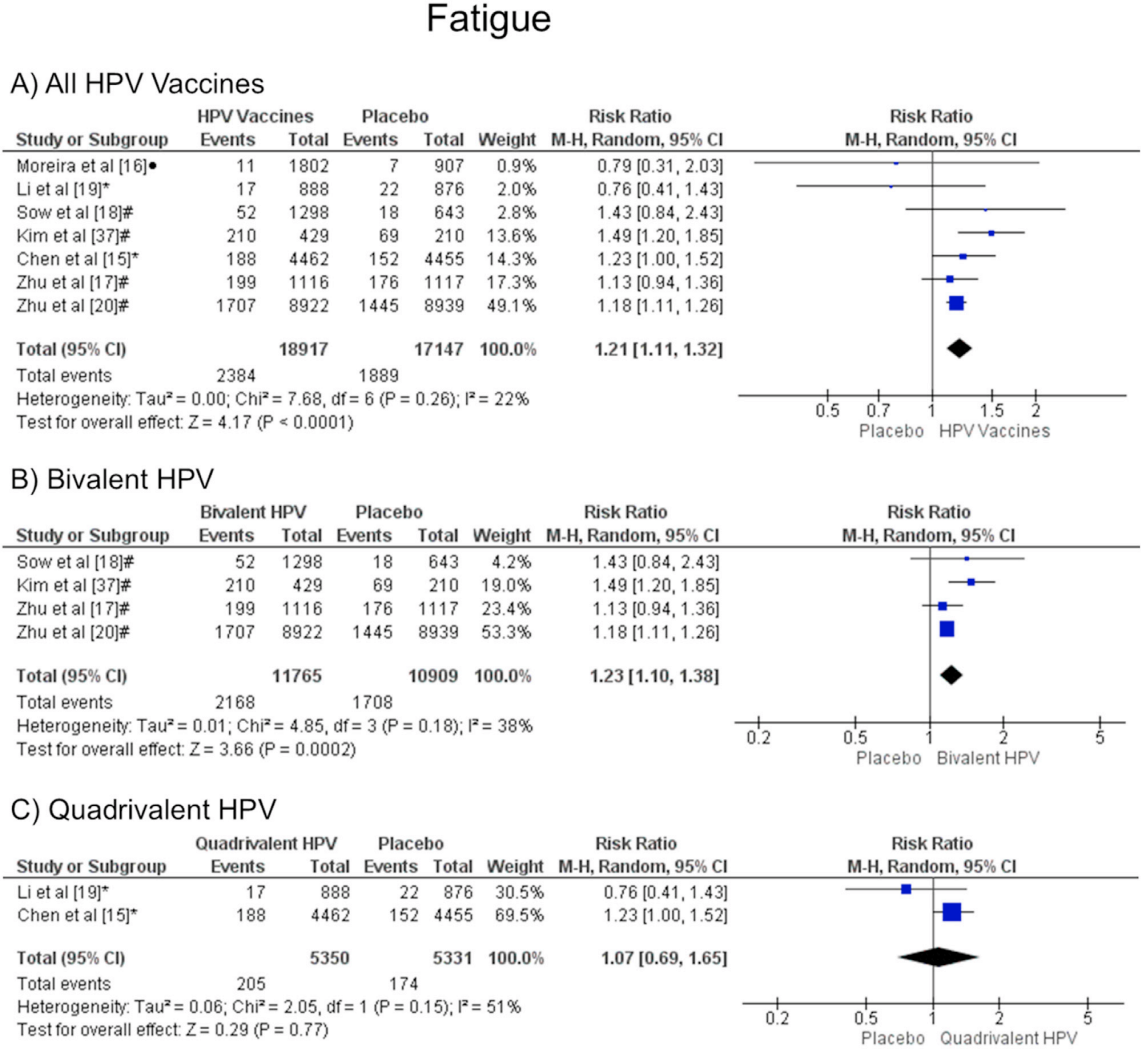
Forest plot of the risk ratio for fatigue symptoms after HPV vaccination. The events indicate how often fatigue symptoms were reported across the three doses, while the total corresponds to the number of participants multiplied by the number of doses. ^a^Bivalent vaccine; ^b^quadrivalent vaccine; ^c^nonavalent vaccine. Funnel plots are presented in Supplementary Figure. 4S.

It was observed that patients vaccinated with the HPV vaccines are more likely to have fatigue when all vaccine valences are grouped (1.21 [CI: 1.11, 1.32]; *p* < 0.0001; I^2^ = 22 %) (Figure. 5a), and when the HPV bivalent vaccine was evaluated (1.23 [CI:1.10, 1.38]; *p* = 0.0002; I^2^ = 38 %) (Figure. 5b). On the other hand, when the quadrivalent vaccine was evaluated, no difference was observed (1.07 [CI: 0.69, 1.65]; *p* = 0.77; I^2^ = 51 %) (Figure. 5c).

### Cutaneous symptoms: rash and urticaria

**Figure. 6:**
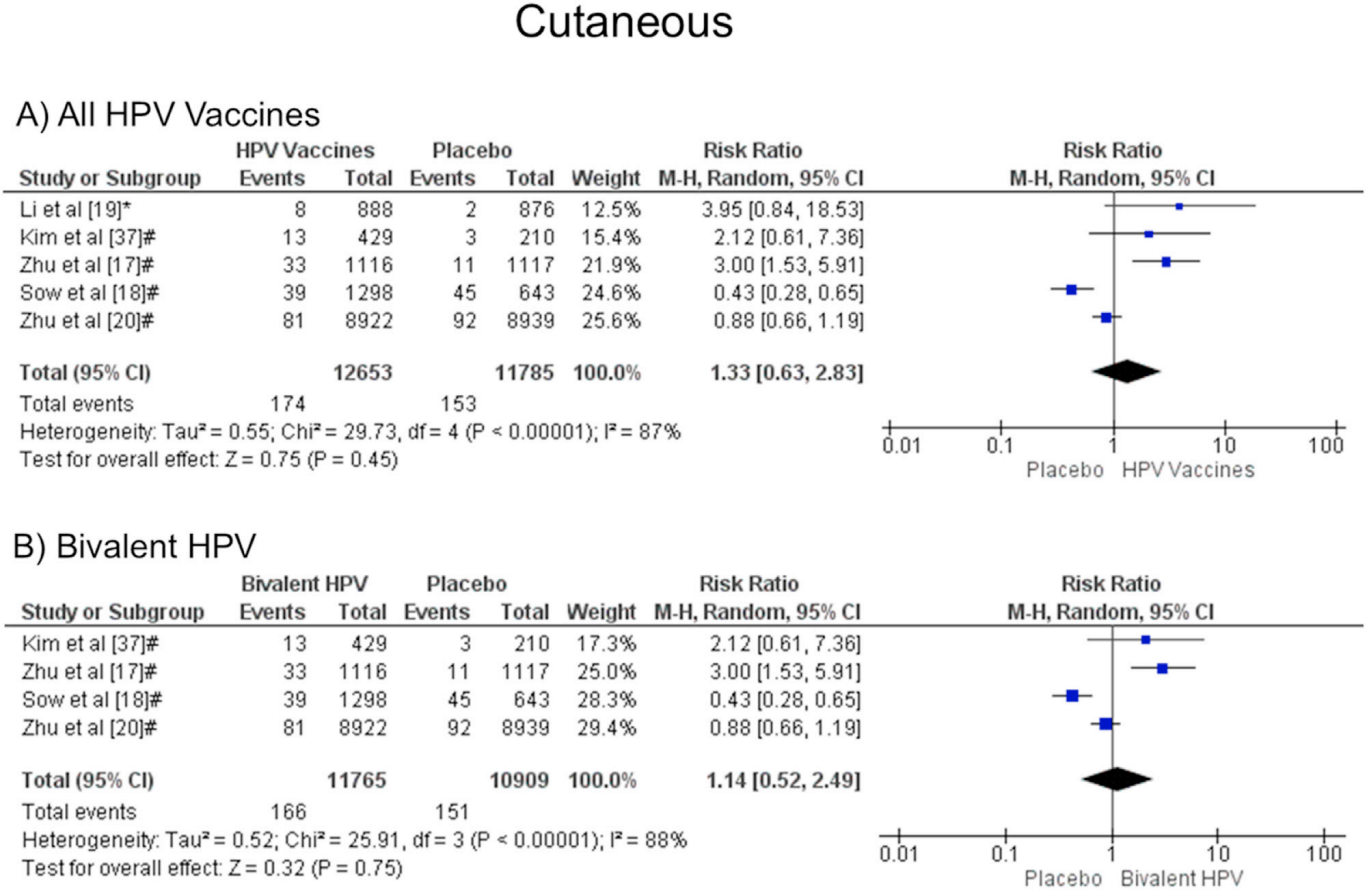
Forest plot of the risk ratio for cutaneous symptoms after HPV vaccination. The events indicate how often cutaneous symptoms were reported across the three doses, while the total corresponds to the number of participants multiplied by the number of doses. ^a^Bivalent vaccine; ^b^quadrivalent vaccine. Funnel plots are presented in Supplementary Figure. 5S.

It was noted that there was no statistical difference in the development of cutaneous symptoms when all vaccine valences were grouped (1.33 [CI: 0.63, 2.83]; *p* = 0.45; I^2^ = 87 %) (Figure. 6a), and when separated by valence, bivalent HPV (1.14 [CI: 0.56, 2.49]; *p* = 0.75; I^2^ = 88 %) (Figure. 6b). Due to the few studies, it was impossible to analyze quadrivalent vaccines separately.

### Myalgia

**Figure. 7:**
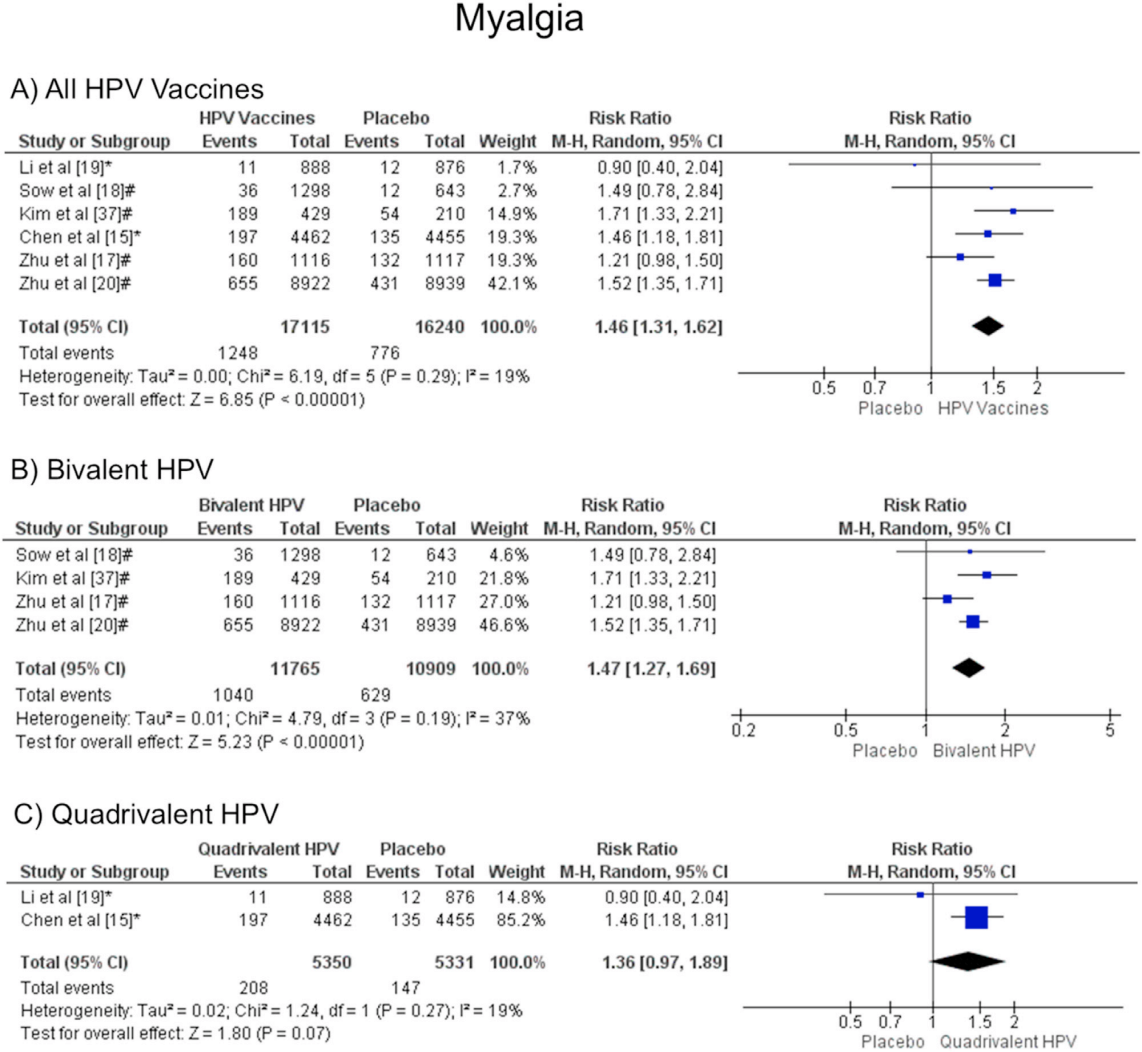
Forest plot of the risk ratio for musculoskeletal pain symptoms after HPV vaccination. The events indicate how often musculoskeletal pain symptoms were reported across the three doses, while the total corresponds to the number of participants multiplied by the number of doses. ^a^Bivalent vaccine; ^b^quadrivalent vaccine. Funnel plots are presented in Supplementary Figure. 6S.

Patients vaccinated with the HPV vaccine are more likely to develop myalgia when all vaccine valences are grouped (1.46 [CI: 1.31, 1.62]; *p* < 0.00001; I^2^ = 19 %) (Figure. 7a), and when separated by valence, bivalent HPV (1.47 [CI: 1.27, 1.69]; *p* < 0.00001; I^2^ = 37 %) (Figure. 7b). On the other hand, when the quadrivalent vaccine was evaluated, no difference was observed (1.36 [CI: 0.97, 1.89]; *p* = 0.07; I^2^ = 19 %) (Figure. 7c).

### Gastrointestinal symptoms: abdominal pain, nausea, vomiting, diarrhea

**Figure. 8:**
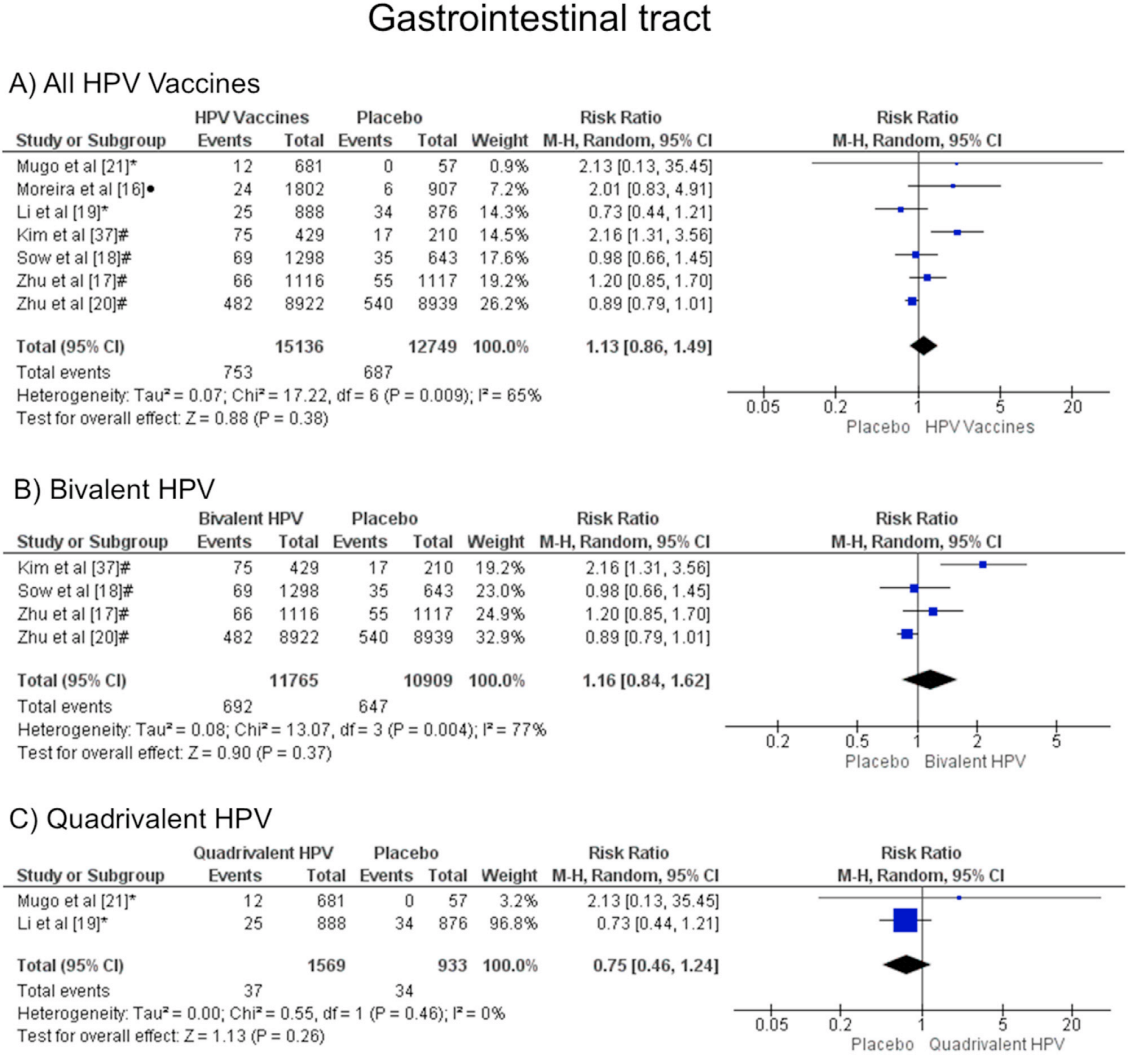
Forest plot of the risk ratio for gastrointestinal tract symptoms after HPV vaccination. The events indicate how often gastrointestinal tract effects were reported across the three doses, while the total corresponds to the number of participants multiplied by the number of doses. ^a^Bivalent vaccine; ^b^quadrivalent vaccine; ^c^nonavalent vaccine. Funnel plots are presented in Supplementary Figure. 7S**.**

When comparing the chances of presenting gastrointestinal symptoms, there was no statistical difference, both groups are likely to present these alterations, when all vaccine valences are grouped (1.13 [CI: 0.86, 1.49]; *p* = 0.38; I^2^ = 65 %) (Figure. 8a), and when separated by valence, bivalent HPV (1.16 [CI: 0.84, 1.62]; *p* = 0.37; I^2^ = 77 %) (Figure. 8b) or quadrivalent (0.75 [CI: 0.46, 1.24]; *p* = 0.26; I^2^ = 0 %) (Figure. 8c).

### Serious adverse reactions vaccine-related

Only three trials reported serious adverse events (SAE) deemed related to vaccination, which precluded the performance of a meta-analysis for this outcome. Chen et al.^15^ identified one SAE–pyrexia occurring three days after administration of dose three in a placebo recipient, which was assessed by the investigator as related to vaccination, the event resolved 29 days after symptom onset. In the study by Moreira et al.,^16^ a single SAE (tonsillitis) was considered by the reporting investigator to be vaccine-related. Zhu et al.^17^ documented one SAE, characterized as a gastrointestinal tract infection, which was deemed possibly related to vaccination by the study investigator.

### Other adverse reactions

Moreover, the studies described other adverse events. Sow et al.^18^ and Li et al.^19^ reported respiratory symptoms after the HPV vaccination and placebo injection. Furthermore, Sow et al.^18^ and Zhu et al.^20^ analyzed the new onset of autoimmune diseases in the control group and the HPV group. Sow et al.^18^ also verified new chronic diseases in both groups. No deaths related to the vaccination were reported across all trials. Due to the limited data, it was impossible to perform a meta-analysis for those outcomes.

### Quality assessment

Overall, all studies were considered excellent on the PEDro scale (9–10 score). Nine of the eleven clinical trials satisfied all the PEDro scale criteria. Since Mugo et al.^21^ was partially double-blind, it had a score of 9 points. Reinsinger et al.^22^ scored 9 points because blinding the therapists who administered the vaccine was not possible (Table 1S).

## Discussion

Vaccine hesitancy, according to the World Health Organization's Strategic Advisory Group of Experts on Vaccine Hesitancy (SAGE-WG), is the delay in accepting or refusing vaccination, despite the availability of vaccination services. This phenomenon, which is multifactorial and difficult to resolve, was identified by the World Health Organization in 2019 as one of the ten main threats to public health.^23^ Thus, as a way of analyzing the reasons for vaccine hesitancy, the SAGE group proposed a model called "3Cs", which refers to three main determinants: Confidence, Complacency, and Convenience. The first factor, trust, refers to knowledge and perceptions about the safety and efficacy of vaccines, taking into account previous experiences of adverse reactions and the credit attributed to the institutions, services, and health professionals involved in the vaccination process. The second factor, complacency, is related to the perception of risk in the face of vaccine-preventable diseases. When individuals do not perceive these diseases as real threats to their health, they consider vaccination unnecessary. This attitude is common in contexts where certain diseases have become rare or have been eliminated, generating a false sense of security. Finally, convenience refers to the ease of access to vaccination services. It involves aspects such as vaccine availability, geographic accessibility, health service opening hours, associated costs (direct and indirect), and language or cultural barriers that may hinder adherence to vaccination.^24^

A cross-sectional analysis of the National Immunization Survey, conducted between 2015 and 2018, revealed a 79.9 % increase in the proportion of parents who refused vaccination against Human Papillomavirus (HPV) for their adolescent children, due to concerns about the safety of the vaccine.^25^ These issues are mainly related to the fear of possible adverse situations. Therefore, this study highlights the main reactions related to the HPV vaccine, clarifying doubts and concerns of patients and caregivers, and demonstrating that the adverse events related to this vaccine are comparable to the side effects associated with other vaccines.

All the trials selected for this study compared HPV vaccines (bivalent, quadrivalent, and nonavalent) to a placebo group. The outcomes most observed in the trials were local reactions, fatigue, myalgia, headache, fever, gastrointestinal symptoms, mucocutaneous alterations, and serious adverse effects. Fatigue and myalgia are more frequent after the HPV vaccination than after the placebo injection. The most common reactions are local site symptoms such as redness, swelling, and pain. Local site reactions were also more frequently observed in those who received HPV vaccines when compared with placebo subjects. There was no significant difference between the HPV vaccine and placebo for the other outcomes evaluated.

The meta-analysis pointed out that people from the HPV vaccine group were more likely to have fatigue when compared to the placebo group (RR 1.21 [95 % CI: 1.11, 1.32]; *p* < 0.0001). The studies analyzed for that outcome presented low heterogeneity (I² = 22 %) according to Higgins et al.^14^ Other reviews showed similar results such as Guo et al.^7^ (RR 1.13 [95 % CI: 1.03, 1.23]; *p* = 0.009), which collected data from 24,031 patients from 8 trials that compared bivalent or quadrivalent HPV vaccines with placebo or other HPV vaccines. Jørgensen et al.^26^ analyzed 95,670 patients of 24 randomized trials that compared 9vHPV, 4vHPV, or 2vHPV with a placebo or a control vaccine, such as the hepatitis A and B vaccines and had similar results to the present work (RR 1.13 [95 % CI: 1.08–1.18], *p* < 0.00001).

When it comes to vaccines against other viruses, fatigue is also more prevalent in the vaccine groups than in the placebo groups. For the COVID-19 vaccine, Sutton et al.^27^ evaluated 223,289 patients and observed differences in the outcomes depending on the type of vaccine - adenovirus vector, inactivated virus, mRNA, and protein subunit. When all kinds of COVID-19 vaccines were analyzed, it was found that patients who received the vaccine were more susceptible to present fatigue than patients in the placebo group (RR 1.69 [95 % CI: 1.59, 1.90], *p* < 0,00,001).

It was observed that myalgia was also more frequent in patients who received the HPV vaccine than in patients who received a placebo. The studies analyzed for this symptom presented low heterogeneity (I² = 19 %). Gonçalves et al.^12^ published similar data for this outcome analyzing four studies that compared the 2vHPV vaccine and placebo (RR 1.97 [95 % CI: 1.77, 2.10]; *p* < 0.00001; I² = 57 %). In addition, Ogawa et al.^28^ found consonant results for myalgia when six trials comparing the 2vHPV vaccine and placebo were observed (RR 1.54 [95 % CI: 1.31, 1.81]). When compared to other vaccines, this outcome is also higher in the vaccine groups than in the placebo groups. Chen et al.^29^ found out that patients who were vaccinated against COVID-19 presented a greater risk of developing myalgia than patients who received a placebo (OR 3.31 [95 % CI: 2.05–5.35]).

This meta-analysis demonstrated that the HPV vaccine group had a greater chance of having local reactions than the placebo group. When separated by valence, the results remained consistent for bivalent and quadrivalent vaccines. Likewise, Huang et al.^30^ showed similar results comparing placebo with 2vHPV (RR 1.16 [95 % CI: 1.09, 1.23]) and with 4vHPV (RR 1.12 [95 % CI: 1.07, 1.16]). That study also observed that injection site events were slightly higher for the 2vHPV vaccine than the 4vHPV (RR 1.60 for the 2vHPV compared to RR 1.31 for the 4vHPV vaccine) when both were compared to the placebo group. This suggests that the 2vHPV vaccine may elicit a stronger local immune response, leading to a higher likelihood of injection site reactions. Additionally, the statistically significant p-values for both vaccines further support the notion that the observed differences are not due to random chance. Therefore, the increased risk ratio associated with the 2vHPV vaccine provides a plausible explanation for the slightly lower incidence of injection site events compared to the 4vHPV vaccine.

For serious adverse events, this study analyzed three trials, which found that these events were rare. Lu et al.^31^ Studies found similar results (RR 1.00 [95 % CI: 0.91, 1.09]) with low heterogeneity (I² = 0 %) for the meta-analysis comparing 2vHPV or 4vHPV with placebo or other vaccines, such as hepatitis A and hepatitis B vaccines.

In a document from the Vaccine Safety Net - a global network of websites organized by the WHO^32^ to provide information on vaccines - on the safety and adverse reactions of the DTP - Diphtheria, Tetanus, Pertussis - vaccine, the main serious adverse reactions of the cellular vaccine, in percentage of occurrences per dose applied, were persistent crying (3.5 %), hypotonic and hyporesponsive episodes (between 0.057 and 0.25 %), convulsions (0.006 %), encephalopathy (between 0.0003 and 0.0053 %) and anaphylaxis (0.0013 %). No p-value was reported.

Another vaccine commonly used for children is the triple viral vaccine against rubella, measles, and mumps. In a retrospective cohort study, Rowhani-Rahbar et al.^33^ evaluated the incidence of fever and convulsions according to the age at which the first dose was administered, concluding that the risk of convulsions is higher for older children, over 15 months old, with RR 6.5 [95 % CI: 5.3, 8.1], with *p* < 0.01. Although serious adverse effects may occur, studies show that they are rare and can occur with other vaccines commonly used in children.

Regarding headache, there was no significant difference between the patients vaccinated against HPV and those who received a placebo. Moreover, Sangar et al.^34^ published similar results for this symptom when four trials analyzing the 2vHPV vaccine and placebo were assessed (RR 0.99 [95 % CI: 0.85, 1.14]; I² = 23 %).

Likewise, the placebo group and the HPV vaccine group had similar risk of developing fever (RR 1.06 [95 % CI: 0.96, 1.16]; *p* = 0.26; I² = 21 %). Coelho et al.^35^ analyzed four clinical trials for the fever outcome, which contrasted placebo and 4vHPV (RD 2 %^1^^,^^3^; *p* < 0.003; I² = 64 %). Therefore, the authors concluded that the vaccines in question are safe and well-tolerated, despite fever being associated with a systemic effect. Setiawan et al.^36^ also observed no significant difference between the HPV vaccine (2vHPV and 4vHPV) and control (placebo or hepatitis A vaccine) for fever events (RR 1.18 [95 % CI: 0.95, 1.48]; *p* = 0.14; I² = 0 %).

Regarding effects on the gastrointestinal tract (RR 1.13 [0.86, 1.49], with *p* = 0.38 and I² = 65 %), there is no statistically significant difference between the effects of the vaccine and placebo. These data are corroborated with other studies, such as Gonçalves et al.,^12^ who indicated values with RR 1.13 [1.00, 1.28], *p* = 0.05 and I² = 70 %, and Ogawa et al.^28^ who, similar to this meta-analysis, there was no significant difference even when the bivalent and tetravalent vaccines were analyzed separately (RR 1.46 [95 % CI: 1.06, 2.02] and RR 0.92 [95 % CI:0.77, 1.11], respectively). However, they had not informed the values of p or heterogeneity to assess whether the results were similar to what was found now: for bivalent, RR 1.16 [95 % CI: 0.84, 1.62], *p* = 0.004 and I² = 77 %, while for tetravalent, RR 0.75 [95 % CI: 0.46, 1.24], *p* = 0.46 and I² = 0.

For cutaneous reactions (RR 1.33 [95 % CI: 0.63, 2.83], *p* = 0.45, and I² = 87 %), there is no statistically significant difference between the effects of the vaccine and placebo. Ogawa et al.^28^ analyzing the bivalent vaccine, separated the reactions in rash and urticaria, and also did not obtain significant differences between vaccine and placebo, with RR 1.26 [95 % CI: 0.80, 1.99] and RR 1.04 [95 % CI: 0.52, 2.08]. In this meta-analysis, five studies brought skin reactions, four with the application of the bivalent vaccine, which obtained RR 1.14 [0.56, 2.49]; *p* = 0.75; I² = 88 %. The above value, with RR 1.33, included the only study with the quadrivalent vaccine that showed such a reaction.

Despite analyzing the study quality using the PEDro tool, to exclude articles with a high risk of bias, great heterogeneity was still obtained in most of the analyses of adverse reactions, especially when vaccines were analyzed separately. Furthermore, few studies were found comparing the 9vHPV vaccine with placebo, making it difficult to analyze the adverse reactions of this vaccine.

## Conclusion

In summary, fatigue and myalgia were more commonly observed in the HPV vaccine group than in the placebo group. Furthermore, patients vaccinated against HPV were more susceptible to developing local reactions when compared to those who received a placebo. There was no significant difference between the HPV vaccine and placebo for the following outcomes: serious adverse effects, gastrointestinal reactions, cutaneous effects, headache, and fever. The HPV vaccine was considered safe in most outcomes, demonstrating a profile of adverse reactions like other vaccines. Since the HPV vaccine prevents some types of cancer, the benefit of applying the vaccine is far greater than the risk.

## Conflicts of interest

The authors declare no conflicts of interest.

## Appendix Supplementary materials

Image, application 1

## Footnotes

Supplementary material associated with this article can be found, in the online version, at doi:10.1016/j.jped.2025.04.009.
