# Supplementary material for: Comparison between the safety of the HPV vaccine versus placebo: a systematic review and meta-analysis of randomized clinical trials
Source: J Pediatr (Rio J). 2025 Jun 6;101(5):101411. doi: 10.1016/j.jped.2025.04.009 (PMC12495592; doi:10.1016/j.jped.2025.04.009)
Supplement: Supplementary file 1 [file mmc1.docx]

**JPED-D-24-00529**

**Supplementary material**

**Comparison between the safety of the HPV vaccine versus placebo: a systematic review and meta-analysis of randomized clinical trials**

**Table 1S** Quality assessment of randomized clinical trial studies about the HPV vaccine versus placebo.

| PEDro scale criteria/ CT | Chen et al.[15] | Kim et al.[37] | Li et al.[19] | Mikamo et al.[11] | Moreira et al.[16] | Mugo et al.[21] | Muñoz et al.[38] | Reinsinger et al.[22] | Sow et al.[18] | Zhu et al.[17] | Zhu et al.[20] |
| --- | --- | --- | --- | --- | --- | --- | --- | --- | --- | --- | --- |
| 1. Eligibility criteria were specified | YES | YES | YES | YES | YES | YES | YES | YES | YES | YES | YES |
| 2. Subjects were randomly allocated to groups (in a crossover study, subjects were randomly allocated an order in which treatments were received) | 1 | 1 | 1 | 1 | 1 | 1 | 1 | 1 | 1 | 1 | 1 |
| 3. Allocation was concealed | 1 | 1 | 1 | 1 | 1 | 1 | 1 | 1 | 1 | 1 | 1 |
| 4. The groups were similar at baseline regarding the most important prognostic indicators | 1 | 1 | 1 | 1 | 1 | 1 | 1 | 1 | 1 | 1 | 1 |
| 5. There was blinding of all subjects | 1 | 1 | 1 | 1 | 1 | 1 | 1 | 1 | 1 | 1 | 1 |
| 6. There was blinding of all therapists who administered the therapy | 1 | 1 | 1 | 1 | 1 | 1 | 1 | 0 | 1 | 1 | 1 |
| 7. There was blinding of all assessors who measured at least one key outcome | 1 | 1 | 1 | 1 | 1 | 0 | 1 | 1 | 1 | 1 | 1 |
| 8. Measures of at least one key outcome were obtained from more than 85% of the subjects initially allocated to groups | 1 | 1 | 1 | 1 | 1 | 1 | 1 | 1 | 1 | 1 | 1 |
| 9. All subjects for whom outcome measures were available received the treatment or control condition as allocated or, where this was not the case, data for at least one key outcome was analysed by “intention to treat” | 1 | 1 | 1 | 1 | 1 | 1 | 1 | 1 | 1 | 1 | 1 |
| 10. The results of between-group statistical comparisons are reported for at least one key outcome | 1 | 1 | 1 | 1 | 1 | 1 | 1 | 1 | 1 | 1 | 1 |
| 11. The study provides both point measures and measures of variability for at least one key outcome | 1 | 1 | 1 | 1 | 1 | 1 | 1 | 1 | 1 | 1 | 1 |
| SCORE | 10 | 10 | 10 | 10 | 10 | 9 | 10 | 9 | 10 | 10 | 10 |


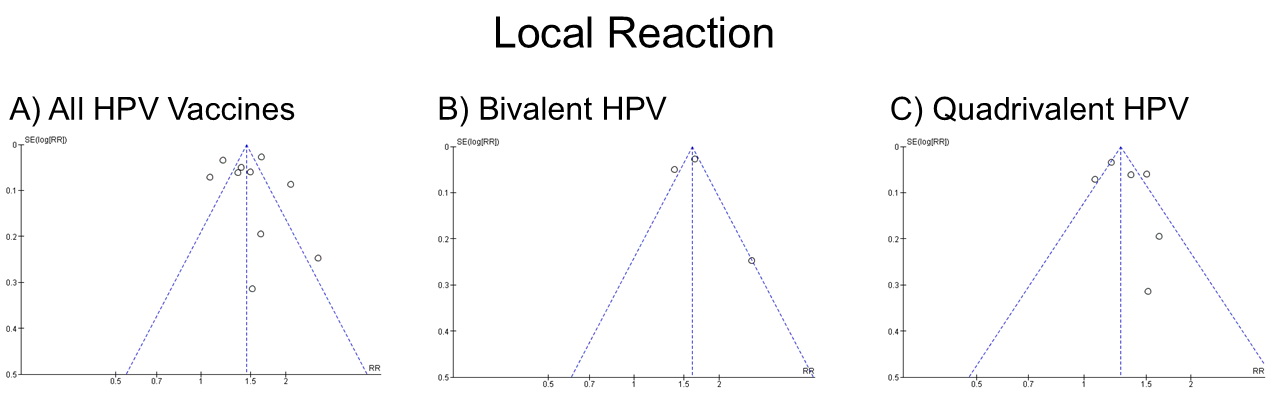


**Figure 1S.** Funnel plot for the local reaction in (A) all HPV vaccines, (B) bivalent HPV and (C) quadrivalent HPV.


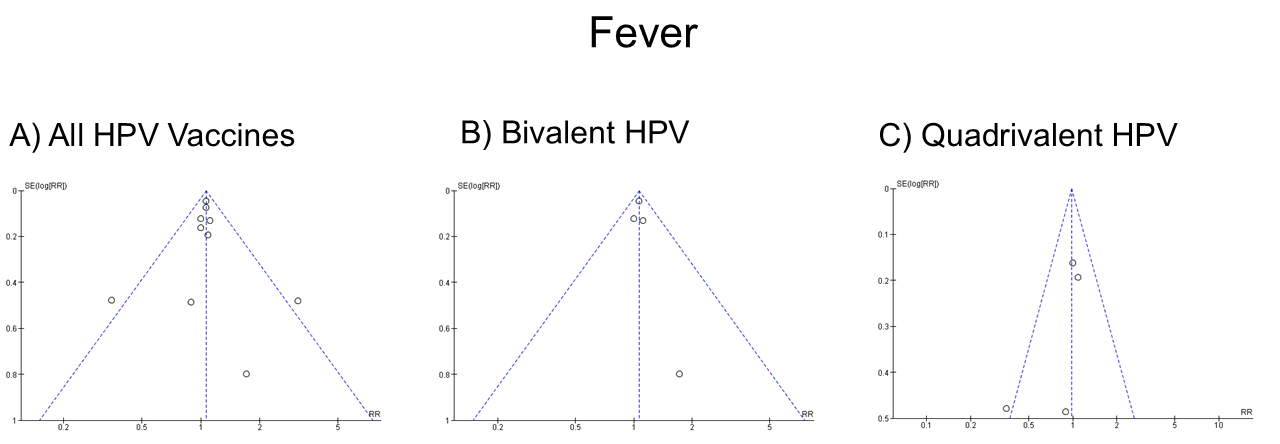


**Figure 2S.** Funnel plot for the fever reaction in (A) all HPV vaccines, (B) bivalent HPV and (C) quadrivalent HPV.


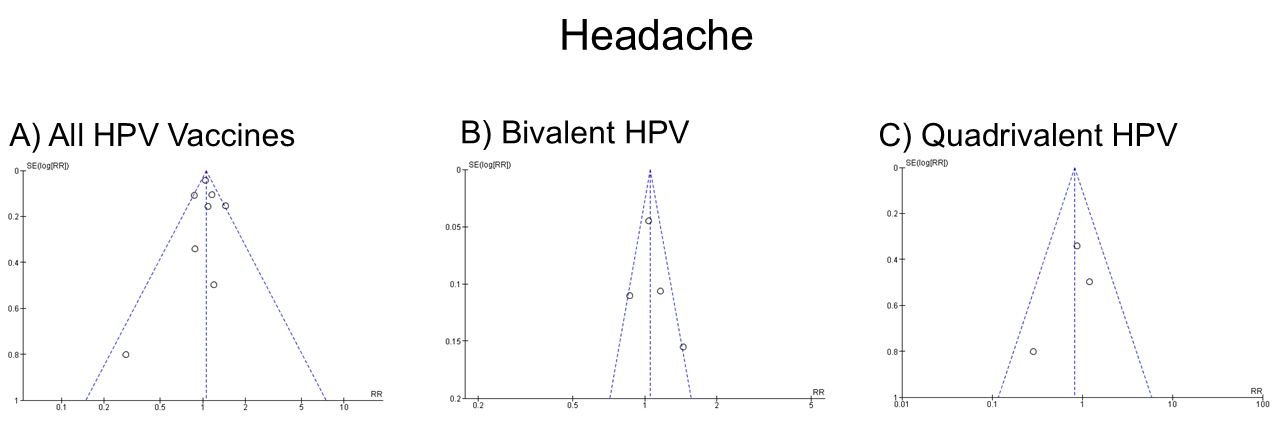


**Figure 3S.** Funnel plot for the headache reaction in (A) all HPV vaccines, (B) bivalent HPV and (C) quadrivalent HPV.


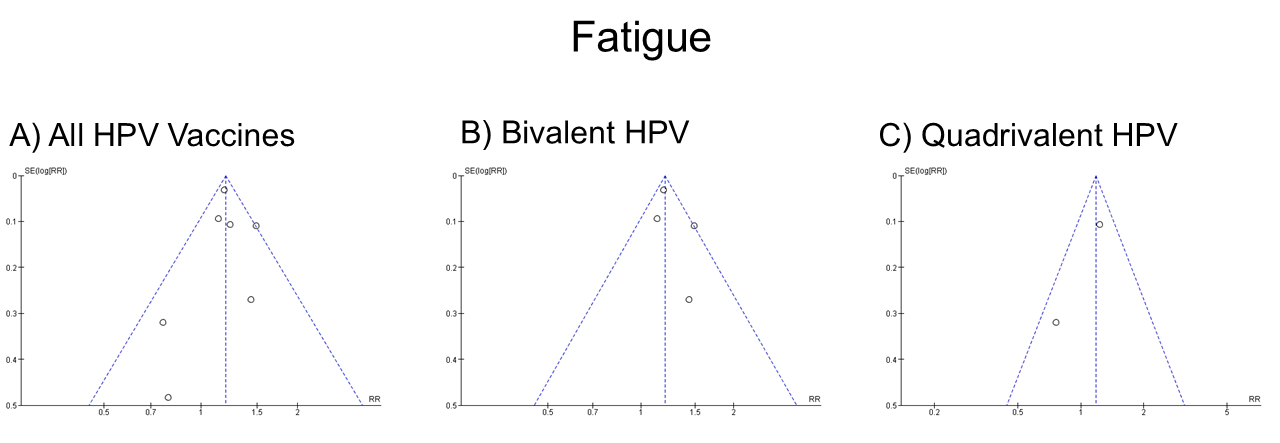


**Figure 4S.** Funnel plot for the fatigue reaction in (A) all HPV vaccines, (B) bivalent HPV and (C) quadrivalent HPV.


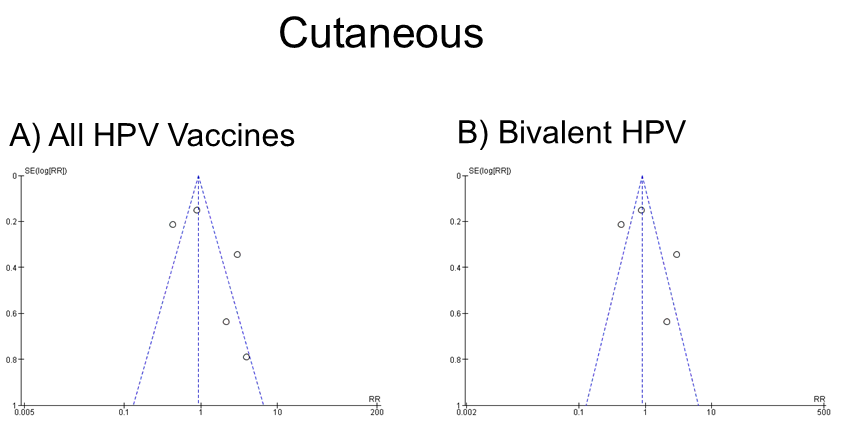


**Figure 5S.** Funnel plot for the cutaneous reaction in (A) all HPV vaccines and (B) bivalent HPV.


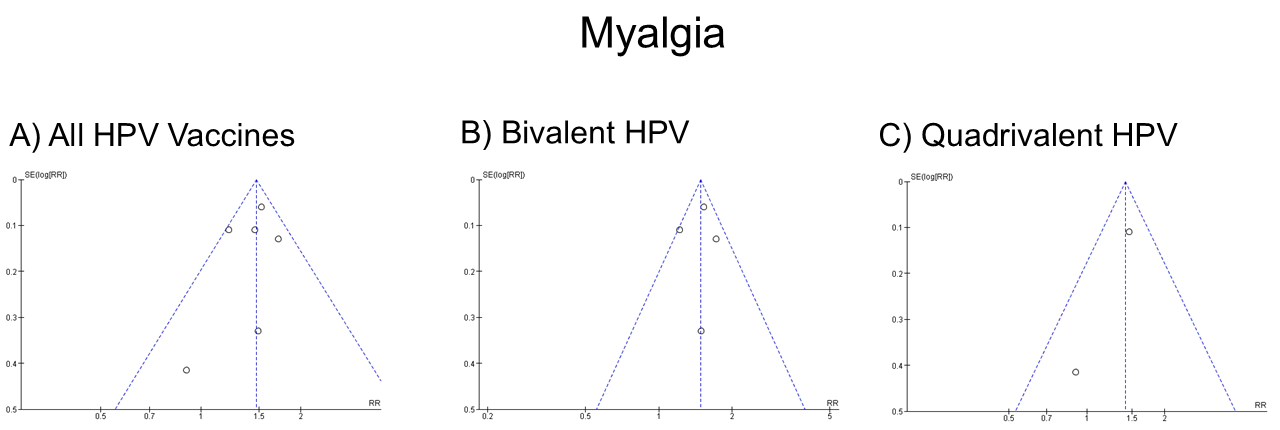


**Figure 6S.** Funnel plot for the myalgia reaction in (A) all HPV vaccines, (B) bivalent HPV and (C) quadrivalent HPV.


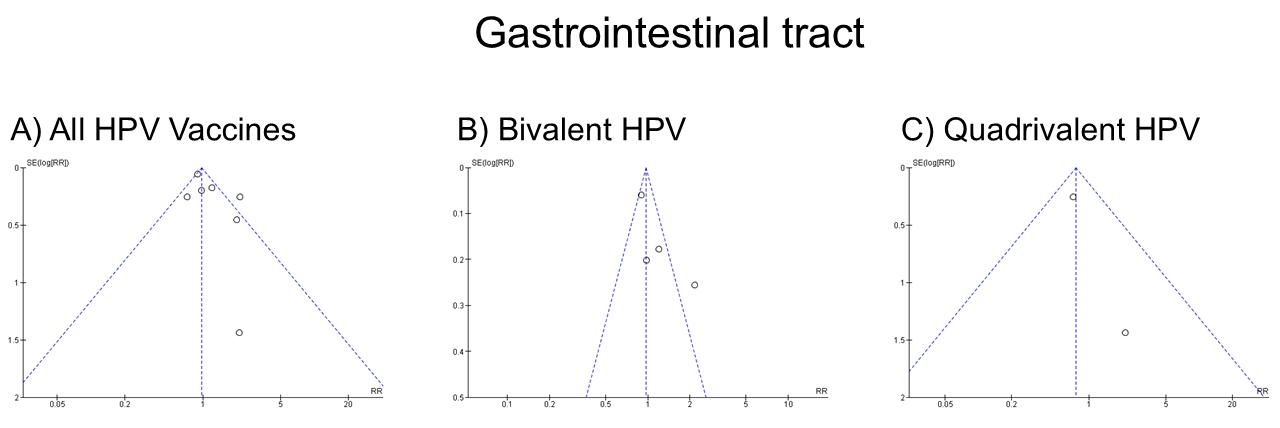


**Figure 7S.** Funnel plot for the gastrointestinal tract reaction in (A) all HPV vaccines, (B) bivalent HPV and (C) quadrivalent HPV.
